# Laser photocoagulation for the treatment of bilateral late-onset retinopathy of prematurity-related retinal detachment in an adult male: A case report

**DOI:** 10.1097/MD.0000000000026227

**Published:** 2021-06-04

**Authors:** Ting Yi Lin, Kathy Ming Feng, Yun Hsiang Chang, I. Chia Liang

**Affiliations:** a Department of Ophthalmology, Tri-service General Hospital, National Defense Medical Center, Taipei; b Ph.D. Program in Nutrition and Food Science, Fu Jen University, New Taipei City, Taiwan.

**Keywords:** laser photocoagulation, retinal detachment, retinopathy of prematurity

## Abstract

**Rationale:**

Retinopathy of prematurity (ROP) is one of the major leading causes of childhood visual morbidity worldwide. Retinal break and traction develop in regressed ROP can further result in rhegmatogenous or tractional retinal detachment years or even decades later.

**Patient concerns:**

Here, we reported a case of bilateral ROP related late complication in a 36-year-old male with a chief complaint of increased floaters in his left eye.

**Diagnoses:**

The fundus examination showed demarcation lines over temporal side in both eyes with tractional retinal detachment and retinal breaks anterior to the lines. A diagnosis of ROP-related late complication of combined tractional and rhegmatogenous retinal detachment was made.

**Interventions:**

Peripheral laser photocoagulation along the demarcation lines for confining the detachment area in both eyes was performed with a stable condition during follow up.

**Outcomes:**

After laser retinopexy, the patient was followed up at one week and four months later with stable laser scars and without progression of the retinal detachments.

**Conclusion:**

Regressed ROP-associated retinal detachment can occur at any time during life. Special care and follow-up may be necessary for these patients.

## 1 Introduction

Retinopathy of prematurity (ROP) is one of the major leading causes of childhood visual morbidity worldwide.^[1]^ Low birth weight, low gestational age, and supplementary oxygen use are the major risk factors.^[2]^ ROP was first reported by Dr. T. L. Terry in 1942, when the intensive neonatal care began to have great improvement.^[3]^ However, it was not until 40 years later that effective treatment for ROP with peripheral retinal ablation therapy was introduced. The age range of the previous premature babies with ROP diagnosed is about 30 to 70 years old. Smith et al reported significant late complications of ROP in adult ROP patients who did not receive treatment as infant.^[4]^ The changes in the regressed phase of ROP could lead to retinal break, traction, and rhegmatogenous or tractional retinal detachment. Previous reports mainly focused on the treatment outcomes of vitrectomy, scleral buckling or both combined.^[4–7]^ We reported a case with good short-term outcome during follow-up after laser retinopexy.

## 2 Case presentation

A 36-year-old male with bilateral hypermetropia, bilateral amblyopia-related poor vision since childhood, and history of strabismus surgery four years ago, presented with increased floaters in his left eye for several days.

**Figure 1:**
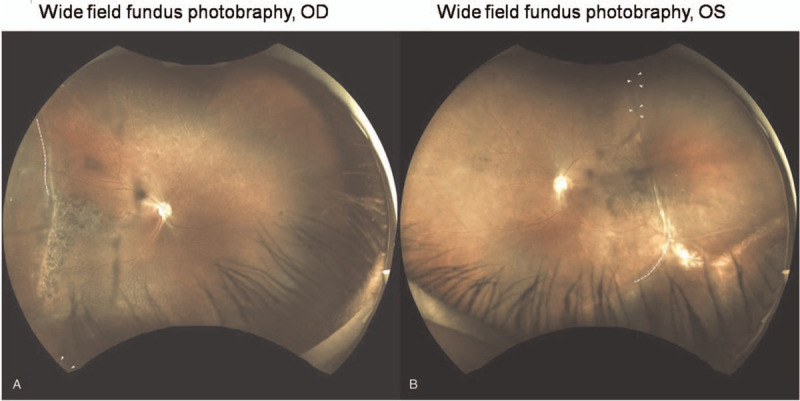
Wide field images at presentation. Both right **(A)** and left eye **(B)** showed demarcation lines over temporal region with pigmentary changes along the border and extended into posterior pole, straightening of the retinal vessels, and tractional retinal detachment with retinal breaks (arrow heads) anterior to the demarcation line. One large break extended from 7 to 8 clock-hours in the right eye and only its upper margin presented in this image (This break presented more thoroughly in Fig. 2). Two spindle-shaped breaks were shown along the demarcation line in the left eye. Barriers of pigmented demarcation bands along the margin of retinal detachment existed except for some areas (dotted lines).

**Figure 2:**
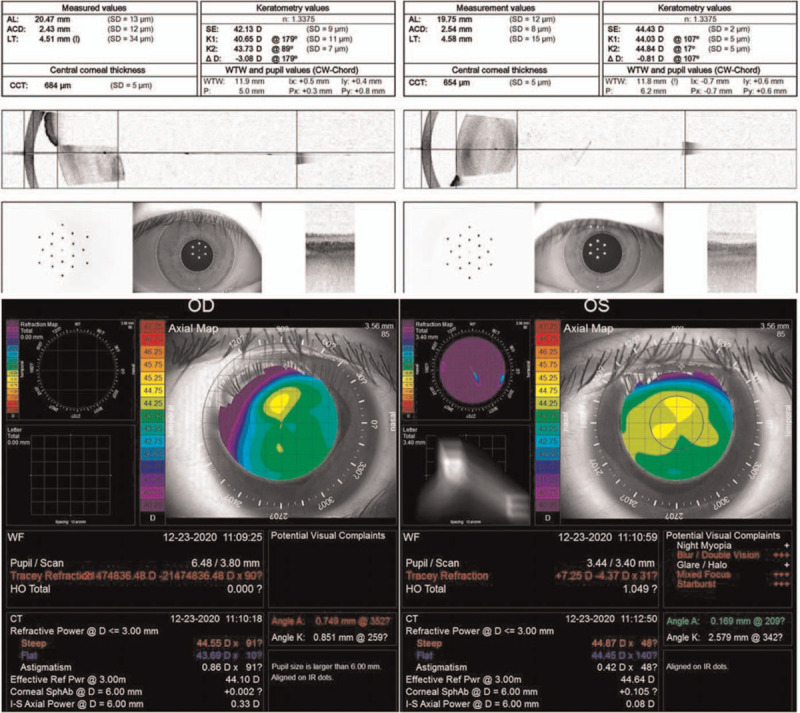
IOLMaster optical biometry (upper) and iTrace wavefront aberrometry examination (lower) showed positive angle kappa in both eyes.

**Figure 3:**
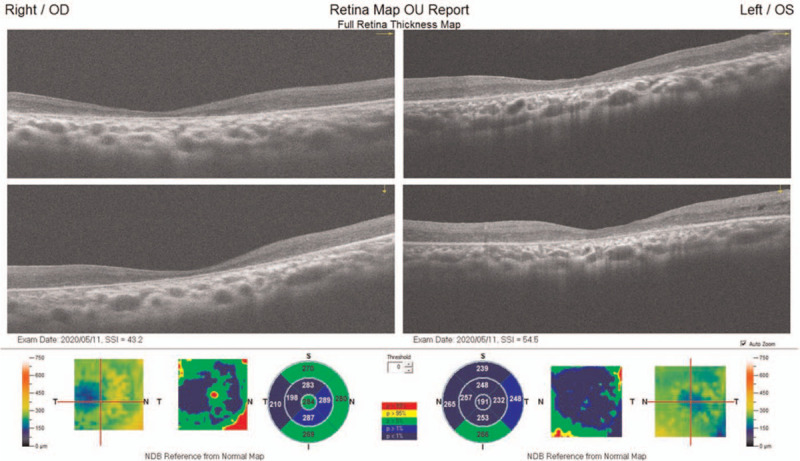
Optical coherence tomography of the macula.

On examination, the best-corrected visual acuity revealed counting fingers in the right eye and 20/200 in the left eye. The intraocular pressure was 14 mmHg in right eye and 9 mmHg in the left eye. Slit-lamp examination revealed mild posterior subcapsular cataract of the left eye. Fundus examination showed bilateral demarcation lines over temporal region with pigmentary boundaries extended into the posterior pole, and straightening of the retinal vessels. Furthermore, tractional retinal detachments with retinal breaks anterior to the demarcation lines were also observed (Fig. 1). He revealed a history of preterm birth at a gestational age of 32 weeks with supplementary oxygen use. External eye examination showed positive angle kappa and this finding was further confirmed by IOLMaster optical biometry and iTrace wavefront aberrometry examination (Fig. 2). Thinning of sensory retina was seen on optical coherence tomography (Fig. 3).

A diagnosis of bilateral ROP-related late complication of delayed combined tractional and rhegmatogenous retinal detachment was made.

Due to the long standing and relatively stable retinal detachment in both eyes, along with the barriers of pigmented demarcation bands that existed along the margins of the detached areas in both eyes, we performed peripheral laser photocoagulation for confining the detachment areas without adequate pigmented demarcation band (dotted lines in Fig. 1).

**Figure 4:**
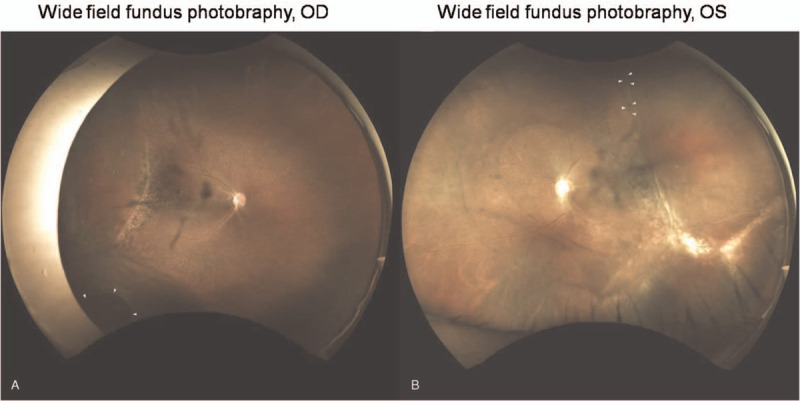
Widefield color fundus photos 4 months after laser retinopexy. **(A)** and **(B)** showed stable condition without progression of the retinal detachments, retinal breaks (arrow heads) or the traction in right and left eyes, respectively.

**Figure 5:**
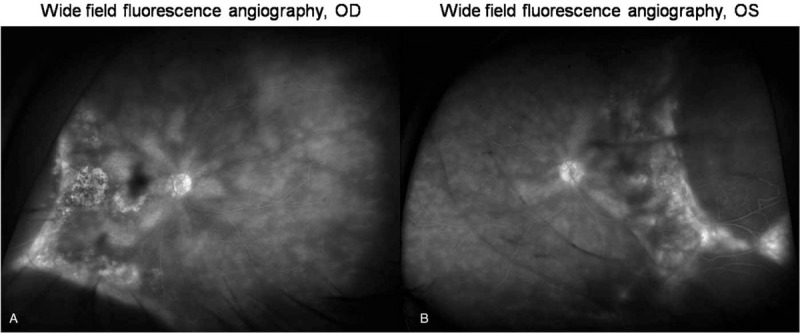
Widefield fluorescence angiography.

After laser retinopexy, the patient was followed up at one week and four months later (Fig. 4) with stable laser scars and without progression of the retinal detachments. Wide field fluorescence angiography revealed the laser scars, pigmented demarcation bands and the ischemia beyond the demarcation lines (Fig. 5)

## 3 Discussion

Several retinal and vascular changes in the regressed ROP patients have been reported, including avascular retina periphery, vascular tortuosity, straightening of blood vessels, retinal pigmentary changes, peripheral retinal folds, lattice-like degeneration, retinal breaks, vitreous membranes, distortion and ectopia of macula, and tractional/rhegmatogenous retinal detachment.^[8]^

A staging system of cicatricial changes in the adult ROP was also reported by Kaiser et al.^[5]^ Clinical fundus features along with preterm history are critical for the diagnosis of adult ROP.

Late-onset retinal detachments in regressed ROP result from the residual and persistent cicatricial vitreoretinal changes. It could be tractional or rhegmatogenous, or the combination of both. Encircling buckle was first reported to treat retinal detachment in regressed ROP in 1966;^[9]^ afterwards, the role of vitrectomy in such retinal detachment was described in 1990.^[10]^ These two techniques or both combined were the main treatment options, even entering the new era of micro incision vitrectomy surgery in the early 2000 s. However, patients with ROP-related retinal detachment were reported to have a relatively low success rate of surgery, even after more than one surgical intervention. One study of 22 eyes with a history of regressed ROP underwent vitreoretinal surgery for rhegmatogenous retinal detachment and showed a low rate of retina reattachment after pars plana vitrectomy or scleral buckle, 46.7% and 71.4%, respectively. Fifty percent eyes showed visual improvement while 27% eyes revealed visual deterioration.^[6]^ Atalay et al also reported that only 64% of the patients who underwent vitreoretinal surgery for cicatricial ROP had complete retinal reattachment in a study which included 11 eyes, among which nine eyes (82%) had visual improvement after complete or partial retinal reattachment.^[7]^ In Park et al's study, the patients with regressed ROP related rhegmatogenous retinal detachment and tractional retinal detachment had an anatomical successful rates of 20% and 54%, respectively, after encircling buckle or vitrectomy and membrane peeling (with or without lensectomy) or both. Visual improvement was seen in two eyes (25%) of the rhegmatogenous retinal detachment group and three eyes (38%) in the tractional retinal detachment group.^[11]^ In another study of 29 eyes described by Tufail et al, the primary anatomical successful rate was 73% in 15 eyes which received encircling buckle, and 57% in the other 14 eyes which required vitrectomy with or without additional buckling. Surprisingly, the final reattachment rate was 97% and 9 eyes received additional intervention between one to three times. However, for macula-on detachments, an overall mean vision loss of one line was observed and 42% of patients had one or more lines of vision loss. For macula-off detachments, there was an overall mean vison gain of one line even though 19% patients had one or more lines of vision loss.^[12]^ The surgical outcomes in these studies implicated the complexity and difficulties in the surgical management of retinal detachment patients with a history of regressed ROP, and pointed out the high risk of vision loss after surgical intervention in macula on situation.

Laser photocoagulation around the margin of subclinical macula-on retinal detachment in eyes with regressed ROP was reported once by Patel et al without details about follow-up or prognosis.^[13]^

We reported a case of good short-term outcome without any significant complications during follow-up after laser photocoagulation reinforcement and suggested that laser photocoagulation reinforcement may be a good treatment option for macula-on retinal detachment related to regressed ROP. This case report highlights the importance of thorough examination and history taking in patients with regressed retinopathy of prematurity. Due to the low primary success rate of surgery and the complex conditions for adult ROP-related retinal detachment, a surgical plan should be made on a person to person basis. Multiple surgeries may be required to achieve retinal reattachment and patients should be informed of this prior to the surgeries. ROP can cause significant late complications to the patients throughout their lifetime and has no correlation with the findings in fundus examinations.^[5]^

This special entity has a historical background in the development of modern neonatology and ophthalmology. Special care and lifelong follow-up may be necessary for these patients.

## Author contributions

**Conceptualization:** Ting Yi Lin, Kathy Ming Feng, Yun Hsiang Chang, I Chia Liang.

**Data curation:** Ting Yi Lin, Kathy Ming Feng, Yun Hsiang Chang, I Chia Liang.

**Formal analysis:** Ting Yi Lin.

**Investigation:** Ting Yi Lin.

**Methodology:** Ting Yi Lin.

**Project administration:** Ting Yi Lin, Yun Hsiang Chang, I Chia Liang.

**Resources:** Ting Yi Lin, Yun Hsiang Chang, I Chia Liang.

**Software:** Ting Yi Lin.

**Supervision:** Yun Hsiang Chang, I Chia Liang.

**Validation:** Ting Yi Lin.

**Visualization:** Ting Yi Lin.

**Writing – original draft:** Ting Yi Lin.

**Writing – review & editing:** Kathy Ming Feng, Yun Hsiang Chang, I Chia Liang.
